# Immunostimulatory and anti-tumor metronomic cyclophosphamide regimens assessed in primary orthotopic and metastatic murine breast cancer

**DOI:** 10.1038/s41523-020-0171-1

**Published:** 2020-07-20

**Authors:** Kabir A. Khan, José L. Ponce de Léon, Madeleine Benguigui, Ping Xu, Annabelle Chow, William Cruz-Muñoz, Shan Man, Yuval Shaked, Robert S. Kerbel

**Affiliations:** 1 Biological Sciences Platform, Sunnybrook Research Institute, Toronto, Canada; 2 Department of Medical Biophysics, University of Toronto, Toronto, Canada; 3 Cell Biology and Cancer Science, Rappaport Faculty of Medicine, Technion, Haifa Israel

**Keywords:** Breast cancer, Chemotherapy, Cancer immunotherapy, Cancer models

## Abstract

The impressive successes of immune checkpoint blockade antibodies to treat various types of cancer are limited to minor subsets of patients. Combination therapy strategies, including with chemotherapy, are being explored to possibly improve the efficacy of immunotherapies. Here we report results regarding the use of an immunostimulatory regimen of metronomic cyclophosphamide (CTX). We show that in orthotopic models of syngeneic murine triple-negative breast cancer (EMT6), CTX administered at 140 mg/kg every 6 days (CTX140 1q6d) is superior at inhibiting primary tumor growth when compared to maximum tolerated dose or daily oral (continuous) low-dose CTX. In SCID or SCID beige mice, anti-tumor effects of CTX140 1q6d are reduced, reinforcing the therapeutic contribution of the adaptive and innate immune systems. In a second breast cancer model (SP1-AC2M2), CTX140 1q6d again showed clear superiority in anti-tumor effects, causing complete tumor regressions; however, these mice were not protected from subsequent tumor re-challenge, suggesting absence of immune memory. We also show that in an aggressive and metastatic cisplatin-resistant variant (EMT6-CDDP), CTX140 1q6d is superior and invokes an influx of intra-tumoral CD4^+^ and CD8^+^ T cells. CTX increases expression of tumor cell PD-L1; however, when combined with concomitant PD-L1 antibody therapy none of the CTX regimens showed increased benefit. This work sheds light on the potential use of metronomic CTX for the treatment of breast cancer, in particular using the quasi-weekly regimen, but also underscores the complexity of the anti-tumor mechanisms and potential to improve immune checkpoint therapy efficacy.

## Introduction

Until recently there were no approved targeted agents for treating the highly aggressive triple-negative breast cancer (TNBC) subtype, which lacks estrogen and progesterone receptors as well as lacking overexpression or amplification of the receptor HER2 (ref. ^1^). This changed with the first TNBC FDA approval of nab-paclitaxel in combination with the PD-L1 antibody atezolizumab^2^. Despite this approval, only a small proportion of patients with metastatic TNBC respond to PD-1/PD-L1 inhibition therapy (approximately 5%)^3^. A possible way to improve these limited immunotherapy benefits include combination with other types of therapy (similar to other types of cancer patients treated with PD-1 or PD-L1 antibodies) such as chemotherapy, vascular endothelial growth factor (VEGF) pathway targeting drugs, or other immune checkpoint inhibitors that ideally do not substantially increase overall toxicity. In principle, one such combination is low or lower dose metronomic chemotherapy, in particular known immunostimulatory regimens of metronomic cyclophosphamide (CTX).

Metronomic chemotherapy using off-patent drugs such as CTX offers potential clinical benefits such as causing less toxicity compared to maximum tolerated dose (MTD) regimens, acting as an immunostimulatory therapy in some cases and being more economically viable especially for use in long-term maintenance therapy settings^4,5^. A recent phase III clinical trial in high-risk pediatric rhabdomyosarcoma patients showed improved survival when vinorelbine and continuous daily low-dose metronomic CTX was used as maintenance therapy after effective induction therapy, leading to this protocol being adopted as a new standard of care for this indication^6^.

Effects of low-dose CTX therapy on cellular components of the immune system have long been known and it has been used as an upfront therapy prior to administration of a tumor vaccine or adoptive immune cell transfer^7,8^. Low-dose daily metronomic cyclophosphamide (LDM CTX) has been shown to reduce numbers of regulatory T cells (T_regs_) preclinically and in patients^9,10^. LDM CTX administered at preclinical doses of 20 mg/kg daily through the drinking water, in mice has been shown to improve survival times in multiple human tumor xenograft models including breast cancer, and could also be combined with other metronomic chemotherapy regimens and/or anti-angiogenic drugs, causing increased overall efficacy^5,11–14^. Daily LDM CTX has also been shown to be a possible combination partner with immunotherapy using CTLA-4 blockade in models of breast cancer^15^. Additionally LDM CTX when combined with vinorelbine and PD-L1 antibodies in preclinical models of breast cancer and lymphoma show additive benefits^16^.

Another schedule of metronomic-like CTX, developed by Waxman and colleagues^17–21^, consisting of 140 mg/kg administered to mice every 6 days (CTX140 1q6d) has shown remarkable efficacy benefits in some preclinical models. This regimen, also known as “medium dose intermittent cyclophosphamide” (MEDIC), has been shown to have potent immunostimulatory effects on natural killer (NK) cells and also result in development of CD8^+^ T cell-mediated complete tumor regressions as well as generation of immune memory in a GL261 glioma model. The CTX140 1q6d regimen has been tested in ectopic models of Lewis lung carcinoma, B16 melanoma, or GL261 glioma implanted subcutaneously as well as a number of subcutaneous implanted human tumor xenografts. CTX140 1q6d was independently shown to enhance survival in orthotopic models of GL261 glioma in comparison to untreated controls^22^. To test whether this regimen of metronomic cyclophosphamide could cause beneficial effects in breast cancer, we employed multiple models of orthotopic syngeneic mouse breast cancer, including a model of adjuvant treatment after surgical resection of primary tumors. The choice of breast cancer as an experimental model for these studies was based on the numerous clinical trials evaluating metronomic chemotherapy in breast cancer patients as an adjuvant or metastatic therapy used alone or in combination with other drugs such as methotrexate, capecitabine, or vinorelbine as well as targeted agents such as bevacizumab and trastuzumab, or an aromatase inhibitor^23–28^. Furthermore, CTX is a commonly used and approved chemotherapy drug for breast cancer, whereas it is not for glioblastoma.

The purpose of the overall studies reported here was to address three related questions. First, what is the comparative effectiveness of three different CTX regimens, one being a maximum tolerated dose (MTD) protocol, a second being low-dose daily/continuous oral metronomic CTX, and the third being the CTX140 1q6d regimen. We report the clear efficacy of the CTX140 1q6d regimen. Second, what is the evidence for immunological mechanisms mediating anti-tumor efficacy of the CTX140 1q6d regimen? We report that both adaptive and innate immunity are involved although this may be breast cancer model dependent, with direct tumor cell targeting effects likely involved in one model tested. Third, would concomitant use of the immune system boosting CTX140 1q6d regimen improve outcomes of PD-L1 antibody therapy? Surprisingly, the answer to this last question was found to be negative, even in a model system where immunostimulatory effects were implicated using the CTX140 1q6d regimen as a monotherapy.

## Results

### CTX140 1q6d extends survival in the EMT6/P mouse breast cancer model

**Fig. 1:**
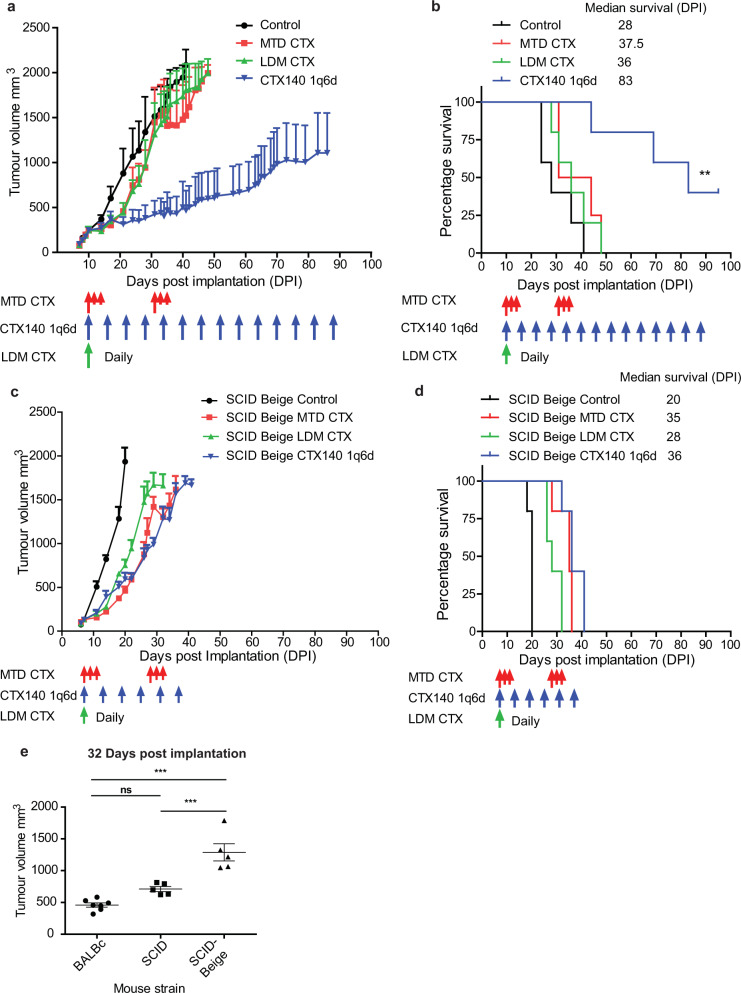
Treatment of EMT6/P orthotopic primary mouse breast cancer with different CTX regimens. **a** Mice bearing EMT6/P were treated with either saline control, MTD CTX (red arrows), LDM CTX continuous daily (green arrow start point), or CTX140 1q6d (blue arrows); data are shown as average tumor growth. **b** Data from **a** of survival. Log rank (Mantel Cox) test Control vs CTX140 1q6d **p = 0.017, *n* = 5 per group. **c** SCID Beige mice bearing EMT6/P were treated with either saline control, MTD CTX (red arrows), LDM CTX continuous daily (green arrow start point), or CTX140 1q6d (blue arrows); data are shown as average tumor growth. **d** Survival data from **c**. **e** Tumor volume comparison at 32 days post implantation in BALB/c wild type, SCID and SCID Beige mice all treated with CTX140 1q6d. Statistical test one-way ANOVA with Bonferroni multiple comparison test ***p < 0.001. Error bars represent SEM.

To determine whether CTX140 1q6d as well as daily continuous LDM CTX or MTD CTX could have effects on orthotopic primary tumor models of syngeneic mouse breast cancer grown in immunocompetent mice, the EMT6/P model was used. Upon treatment of established EMT6/P tumors in BALB/c mice CTX140 1q6d significantly reduced the rate of tumor growth in comparison to saline control-treated mice and had clearly superior effects when compared to either the LDM CTX or MTD CTX regimens (Fig. 1a). When tumor endpoint was used as a surrogate for survival CTX140 1q6d increased survival significantly over control, whereas MTD CTX and LDM CTX failed to do so (Fig. 1b). To determine whether the anti-tumor effects of CTX140 1q6d were caused by, at least in part, immunological effector cells, EMT6/P was implanted into severe combined immunodeficient (SCID) Beige mice which lack functional T cells, B cells, and NK cells^29^. The results clearly showed that with each CTX regimen, there were temporary anti-tumor effects at the beginning of treatment (likely due to the direct anti-proliferative or cytotoxic effects on the tumor cells). The benefit or extension in survival observed in immunocompetent mice treated with CTX140 1q6d (Fig. 1a, b) was lost in the SCID Beige mice (Fig. 1c, d).

To determine whether a lack of T cells (or B cells) would nullify or compromise the potent anti-tumor effects of the CTX140 1q6d regimen, SCID mice which have functional NK cells but lack functional T and B cells were implanted with EMT6/P and treated with CTX140 1q6d (Supplementary Fig. 1). When tumor volumes were compared between each mouse type bearing EMT6/P and treated with CTX140 1q6d, there was a trend in decreased efficacy in SCID mice but a significant difference between BALB/c mice compared to SCID Beige (Fig. 1e). The time point of 32 days post implantation was chosen for analysis as this was the last time point in which all mice in each group treated with CTX140 1q6d were still present. To further compare the differences between each mouse strain, survival analyses were performed on control-treated and CTX140 1q6d-treated mice (Supplementary Fig. 1). This analysis revealed a significant difference in survival of control-treated mice between both SCID and SCID Beige when compared with normal BALB/c mice; however, in control-treated animals there was no significant difference in survival between SCID and SCID Beige mice, suggesting that NK cells may not play a prominent role in affecting the growth of this tumor model in untreated mice. However, in the CTX140 1q6d-treated groups, there were significant differences between all mouse strains including between SCID and SCID Beige, suggesting that NK cells contribute to the therapeutic efficacy of the CTX140 1q6d regimen in this model. As a surrogate for toxicity mouse weights were recorded which showed that in all CTX treatments mice gained weight but not at the same rate as control-treated mice (Supplementary Fig. 2).

### CTX140 1q6d extends survival in SP1-AC2M2 mouse breast cancer model

**Fig. 2:**
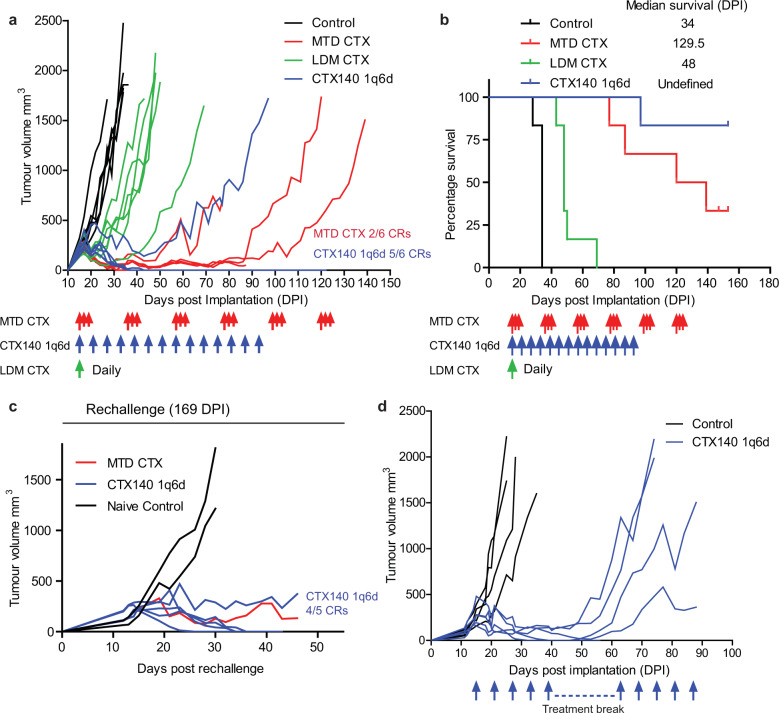
Treatment of SP1-AC2M2 orthotopic primary mouse breast cancer with different CTX regimens. **a** CBA/J mice bearing SP1-AC2M2 tumors were treated with either saline control, MTD CTX (red arrows), LDM CTX continuous (green arrow at the start point) or CTX140 1q6d (blue arrows). Two of six complete responses were observed in MTD CTX and 5/6 complete responses in CTX140 1q6d. **b** Survival data from **a** when mice reached tumor endpoint as a surrogate for survival. Log rank (Mantel Cox) test **p < 0.01 for all groups when compared to control treatment *n* = 6 per group. **c** Mice displaying complete responses were re-challenged with SP1-AC2M2 in the opposite mammary fat pad to the original implantation. All mice that displayed completely regressed tumors grew tumor re-challenges and were then treated with the original CTX therapy resulting in 4/5 complete responses when treated with CTX140 1q6d. **d** SP1-AC2M2 primary tumors were treated with CTX140 1q6d (blue arrows), a break was initiated after five doses. This approach did not induce complete responses and tumors were then non-responsive to CTX140 therapy after re-initiation.

To determine whether similar effects observed in the EMT6/P breast cancer model could be recapitulated in a different mouse breast cancer model and in a different background mouse strain, the SP1-AC2M2 cell line implanted into the mammary fat pad of CBA/J mice was utilized^30,31^. This highly aggressive breast cancer cell line was derived from metastatic lung nodules after three serial passages in syngeneic CBA/J mice using the cell line called SP1 (which itself arose spontaneously in an aged CBA/J mouse^30^). This primary tumor model was much more sensitive to each regimen of CTX when compared to EMT6/P (Fig. 2a). Thus, the daily continuous LDM CTX treatment resulted in significant extension of survival (Fig. 2b), whereas MTD CTX and the CTX140 1q6d treatment protocol resulted in far superior therapeutic effects (Fig. 2a, b). In particular, CTX140 1q6d resulted in 5/6 complete responses (CRs) whereas MTD CTX resulted in 2/6 CRs (Fig. 2a). These CRs were achieved after 5–6 doses of CTX140 1q6d or one cycle of MTD CTX. Each CTX treatment regimen was ceased when tumors became undetectable. The CTX140 1q6d and MTD CTX regimens clearly prolonged survival times of mice in this breast cancer model (Fig. 2b). In order to determine whether these complete responses resulted in initiation of an immune memory, mice displaying CRs were re-challenged with the same cell line in the inguinal mammary fat pad on the opposite flank. This resulted in growth of tumor re-challenge at a rate comparable to naïve mice indicating a lack of immune memory (Fig. 2c). When tumors from these re-challenged mice reached a size of 200–250 mm^3^, the original CTX therapy protocol was resumed, showing these tumors remained responsive, resulting in 4/5 complete regressions with CTX140 1q6d; however, one tumor re-challenge treated with CTX140 1q6d failed to completely respond (Fig. 2c).

To determine whether CTX140 1q6d treatment could result in CRs after treatment cessation due to a lasting immune response, a drug break was initiated after five doses of CTX140 1q6d in the SP1-AC2M2 model (Fig. 2d). Upon treatment cessation no CRs were observed, and tumors began to increase in size; interestingly, upon retreatment with CTX140 1q6d, beginning at 63 days post implantation (DPI) these tumors were completely unresponsive (Fig. 2d). This suggests that direct tumor cell killing effects are needed in constant 6-day intervals in this model to successfully clear the tumor along with its likely immunostimulatory effects.

These results suggest that the major anti-tumor effects of CTX in this model stem from the cytotoxic activity of the drug and if there is an immune cell component, it is likely related to the innate immune system.

### CTX140 1q6d extends survival and increases CD4^+^ and CD8^+^ T cell number in a cisplatin resistant and aggressive variant of EMT6

**Fig. 3:**
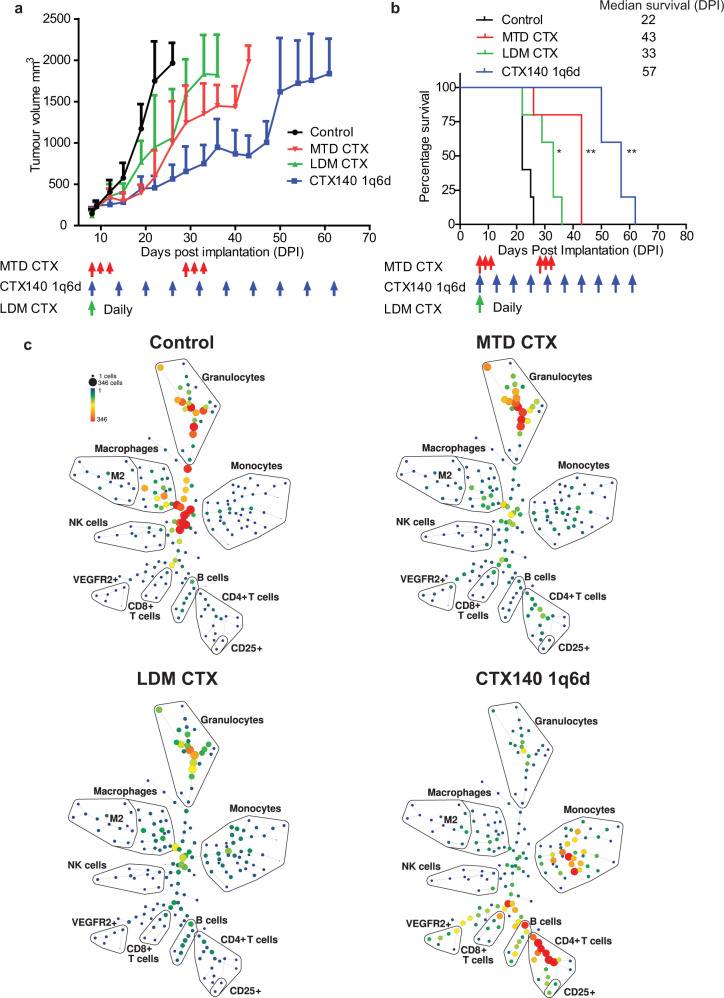
Treatment of EMT6-CDDP orthotopic primary mouse breast cancer with different CTX regimens. **a** Mice bearing EMT6-CDDP were treated with either saline control, MTD CTX (red arrows), LDM CTX continuous daily (green arrow start point), or CTX140 1q6d (blue arrows). **b** Survival data from **a**. Log rank (Mantel Cox) tests; CTX140 1q6d vs control **p = 0.0015, MTD CTX vs control **p = 0.0041, LDM CTX vs control *p = 0.016, *n* = 5 per group. **c** The EMT6-CDDP primary tumor treatment experiment was repeated and tumors were analyzed by CyTOF at 29 DPI (18 days after the start of treatment), i.e., after one cycle of MTD CTX and three doses of CTX140 1q6d. CyTOF analysis of four pooled tumors from each treatment group. SPADE trees of each treatment group, the size of each sphere represents number of cells, and the color represents expression level of CD45. Error bars represent SEM.

To determine the effects that each CTX regimen has on a more aggressive and metastatic murine TNBC line, the EMT6-CDDP model was used. This cell line was originally generated and selected as an in vivo variant of EMT6 resistant to repetitive high-dose cisplatin therapy (CDDP)^32^. CTX140 1q6d significantly reduced tumor growth in comparison to control treatment and extended survival (Fig. 3a). In order to analyze changes in the tumor immune microenvironment after each CTX regimen in this tumor model, time of flight mass cytometry (CyTOF) was used to analyze the expression of 37 different cell markers simultaneously. Mice bearing EMT6-CDDP tumors were treated with one cycle of MTD CTX, or three doses of CTX140 1q6d, or continuously treated with LDM CTX, 29 days post implantation or after 18 days of treatment, mice were sacrificed, and tumors analyzed by CyTOF (Fig. 3b, Supplementary Figs 3 and 4). The results revealed an increase in B cells, CD4^+^ T cells, and CD8^+^ T cells in the CTX140 1q6d group whereas such striking increases were not observed in the other two treatment groups at this time point (Fig. 3c). The CTX140 1q6d regimen also reduced granulocyte populations while increasing monocytes. Importantly, when memory markers CD44 and CD62L were analyzed on CD4^+^ and CD8^+^ T cells to identify central memory cells (CD44^+^, CD62L^−^) or effector memory cells (CD44^+^, CD62L^−^), reductions were observed in each CTX treatment group compared to control, with the largest reduction in the CTX140 1q6d group (Supplementary Fig. 3).

### Adjuvant treatment of CTX regimens in postsurgical metastasis models of EMT6-CDDP

**Fig. 4:**
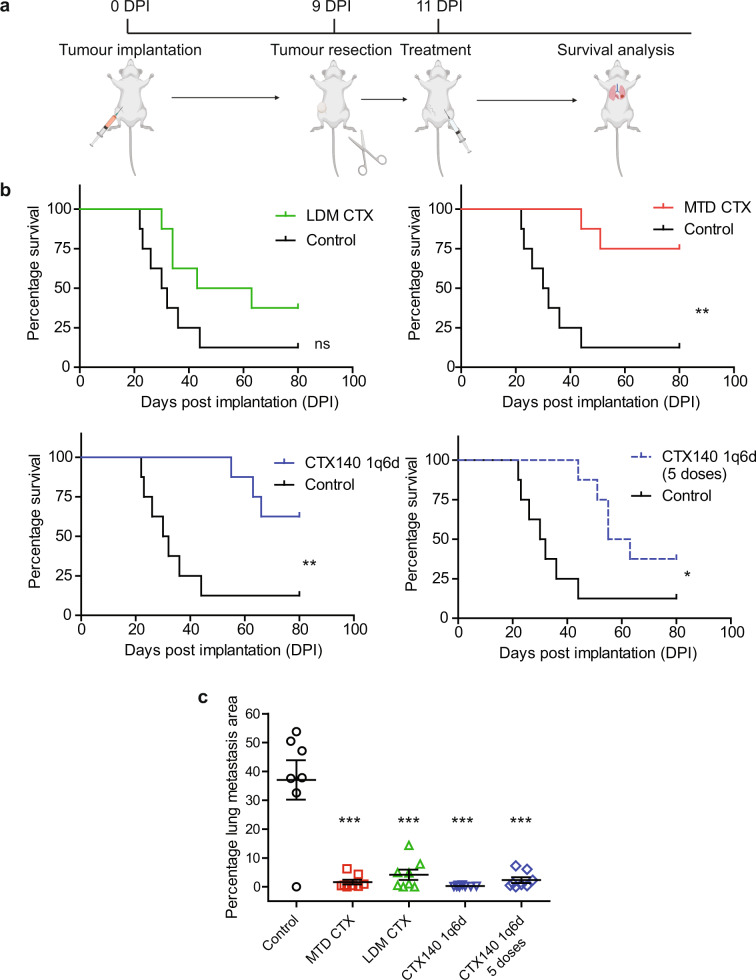
Postsurgical adjuvant CTX treatment in EMT6-CDDP model. **a** Schematic diagram of the experimental method created with Biorender.com. **b** Survival analyses of each treatment group plotted with the same control treatment group for comparison. Log rank (Mantel Cox) **p < 0.01, *p < 0.05, *n* = 8 per group. **c** Analysis of percentage lung metastasis visualized by immunohistochemical staining using Ki67 as a marker for proliferation. Statistical test one-way ANOVA with Bonferroni multiple comparison test ***p < 0.001. Error bars represent SEM.

To determine whether any of the CTX regimens could prolong survival times when administered in the postsurgical adjuvant treatment setting, the metastatic cell line model EMT6-CDDP was utilized. Mice were implanted with EMT6-CDDP in the mammary fat pad and subsequently the primary tumors were resected upon reaching approximately 200 mm^3^. Two days after resection each CTX therapy regimen was initiated (Fig. 4a). In order to test whether CTX140 1q6d would need to be given continuously to have beneficial effects in this treatment setting, one group of mice was treated with five doses and another was treated with continuous CTX140 1q6d. This resulted in an extension of survival with MTD CTX and both CTX140 1q6d treatments. Experiment endpoint was reached when mice displayed labored breathing (a symptom of high lung metastasis burden), weight loss over 20%, or regrowth of the resected primary tumor reaching 1700 mm^3^. Control-treated mice reached endpoint due to labored breathing and displayed macroscopic metastases in seven out of eight mice (Supplementary Fig. 5a). Interestingly, none of the MTD CTX or CTX140 1q6d treatment groups displayed such labored breathing and endpoint was reached due to primary tumor regrowth. Survival analyses revealed no significant difference between control and LDM CTX treatments, whereas there was a significant difference in survival between MTD CTX, continuous CTX140 1q6d, and CTX140 1q6d (five doses) when compared to control (Fig. 4b, Supplementary Fig. 5b). To determine whether any of the CTX regimens had an effect on the level of lung metastatic burden, endpoint lung samples were analyzed for metastatic nodules by staining for Ki67. A dramatic reduction in lung metastases was observed in all CTX treatment groups when compared to control, with the largest reduction in CTX140 1q6d (Fig. 4c, Supplementary Fig. 5c).

### CTX increases expression of PD-L1 in mouse breast tumor cell lines

**Fig. 5:**
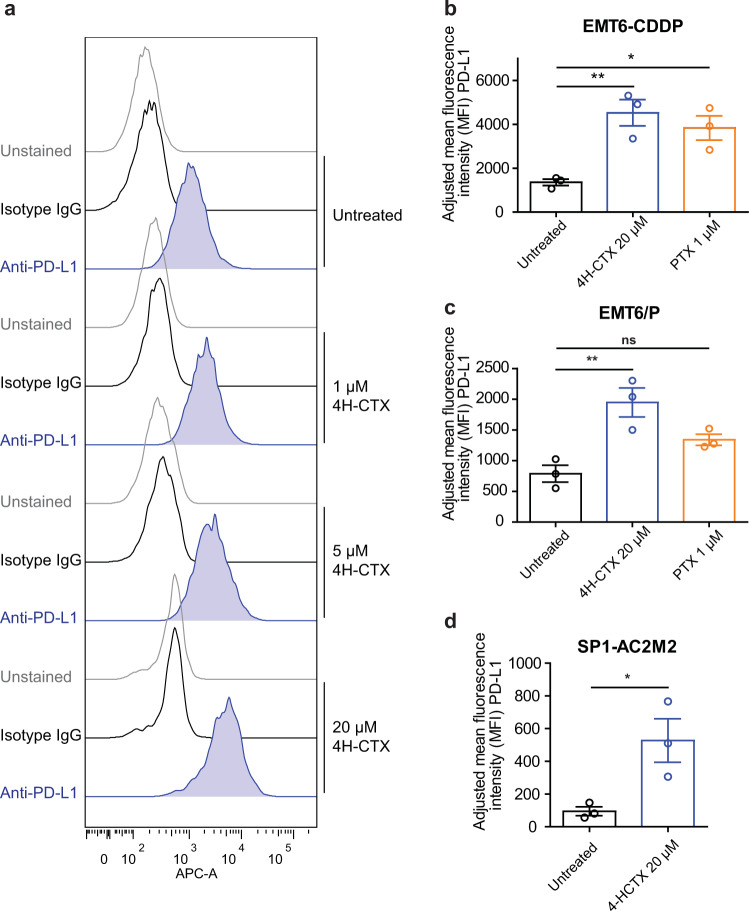
PD-L1 expression increases in response to 4H-CTX or paclitaxel (PTX) treatment in vitro. **a** EMT6-CDDP cells were treated with increasing concentrations of 4H-CTX, which resulted in increased expression of PD-L1 analyzed by flow cytometry. **b** EMT6-CDDP, **c** EMT6/P, or **d** SP1-AC2M2 cells were treated with 20 µM 4-HCTX or 1 µM of PTX; values represent mean fluorescence intensity of PD-L1 analyzed by flow cytometry with subtraction of mean fluorescence intensity of isotype IgG. One-way analysis of variance with Tukey’s multiple comparison test. Unpaired *t*-test for SP1-AC2M2 analysis. *p < 0.05, **p < 0.01. Error bars represent SEM.

It has been previously shown that a range of different chemotherapeutic agents can upregulate the expression of various immune checkpoint molecules such as PD-L1, which may have effects on chemoresistance as well as enhancing tumor evasion of the immune system in breast cancer^33,34^. EMT6/P, EMT6-CDDP, and SP1-AC2M2 cells were treated with the active toxic metabolite form of CTX, 4-hydroperoxy cyclophosphamide (4H-CTX), in vitro in order to test whether CTX treatment can result in increased cell surface expression of PD-L1. Such in vitro treatment did indeed increase the cell surface expression of PD-L1 in a concentration-dependent manner (Fig. 5, Supplementary Fig. 6a). Paclitaxel (PTX) was included as a positive control for a chemotherapy drug previously shown to upregulate PD-L1 (ref. ^33^) (Fig. 5, Supplementary Fig. 6). Additionally, PD-L1 expression was found to be increased in vivo after three doses of CTX140 1q6d on SP1-AC2M2 tumor cells (CD45^−^) as well as CD45^+^ leukocytes in comparison to cells from saline control-treated tumors (Supplementary Fig. 7). Interestingly, PD-L1 expression was reduced on CD45^+^ cells when splenocytes were analyzed from CTX140 1q6d-treated mice in comparison to control-treated mice.

### PD-L1 blockade does not increase efficacy of CTX therapy in EMT6-CDDP model

**Fig. 6:**
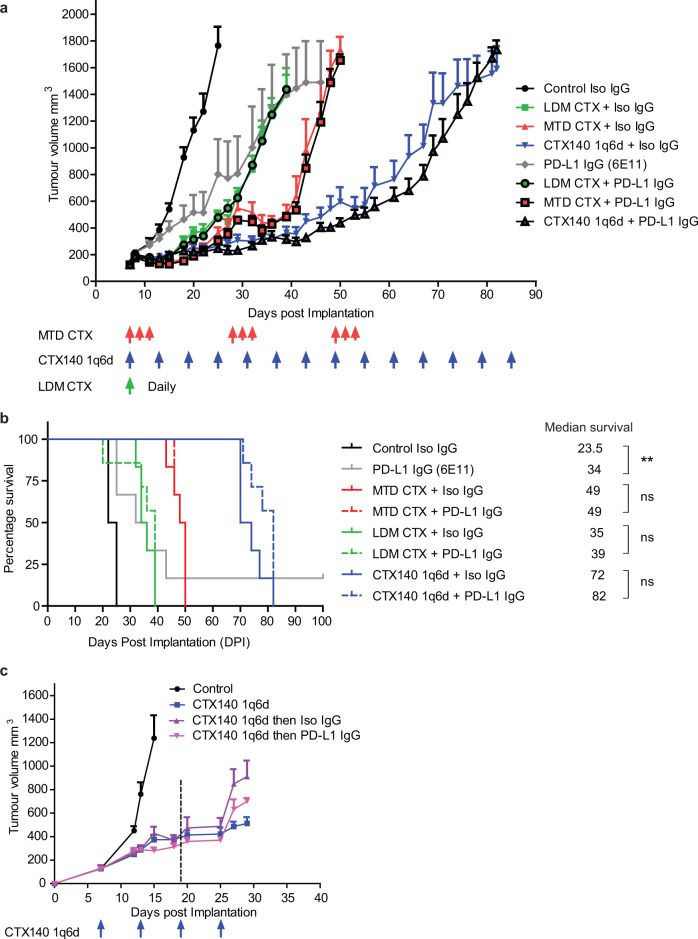
CTX in combination with PD-L1 blockade does not synergize in primary EMT6-CDDP models. **a** Primary tumor growth of EMT6-CDDP treated concomitantly with either IgG isotype control or PD-L1 blockade in combination with each regimen of CTX or saline control. **b** Survival analysis of data from **a**. Log rank (Mantel Cox) test **p = 0.0088, *n* = 6 per group. **c** Induction therapy with CTX140 1q6d then followed up with either IgG isotype or anti-PD-L1 in the EMT6-CDDP model. Dotted line denotes when CTX140 1q6d treatment was ceased in both IgG Isotype and anti-PD-L1 groups. Error bars represent SEM.

We have previously shown that PD-L1 blockade can have therapeutic effects in the EMT6-CDDP tumor model when treating both primary tumors and postsurgical metastatic disease in the neoadjuvant or adjuvant treatment settings^35,36^. Due to the findings of increased PD-L1 expression after CTX treatment, as well as CyTOF data suggesting that there was an increased infiltration of CD4^+^ and CD8^+^ T cells seen with CTX140 1q6d therapy, this model was used for testing whether any of the CTX regimens in combination with PD-L1 blockade could enhance overall therapeutic effects. Therefore, each CTX regimen was combined with a preclinical surrogate version of atezolizumab, a mouse antibody raised against mouse PD-L1 (clone 6E11) in comparison to a mouse isotype control IgG^36,37^. The antibodies and chemotherapy treatments were administered concomitantly. No significant benefit in any of the combination treatment groups was observed (Fig. 6a). PD-L1 blockade monotherapy had modest anti-tumor effects similar to results obtained previously and significantly extended survival^35^ (Fig. 6b). Next, upfront or induction CTX140 1q6d therapy was employed to test whether this could stimulate an immune response by enhancing immunogenic cell death and increasing infiltration or expansion of T cells within the primary tumor, which could then be followed up with PD-L1 blockade therapy. Three doses of CTX140 1q6d was chosen as an upfront regimen as this was the time point in which CD4^+^ and CD8^+^ T cells were present as shown by CyTOF analyses. CTX140 1q6d was ceased in two groups of mice at 19 DPI and then treated with either isotype IgG or PD-L1 antibody, another group of mice continued to receive CTX140 treatment. This resulted in no significant difference between IgG or PD-L1 treatment after upfront CTX140 1q6d and there appeared to be a trend of benefit when mice were continuously treated with CTX140 1q6d alone (Fig. 6c).

## Discussion

The primary goal of this study was to comparatively evaluate three different CTX protocols for anti-tumor efficacy in three highly translatable murine breast cancer models with particular emphasis on an every 6-day metronomic-like regimen using a dose of 140 mg/kg (CTX140 1q6d). Previous studies indicated CTX140 1q6d has potent stimulating effects on both the innate and adaptive immune systems^18,19^. As such, we considered CTX140 1q6d as a potentially ideal partner for improving immune checkpoint antibody therapy, which was assessed as a secondary goal of these studies. Among the more interesting results we obtained include: (i) CTX140 1q6d is clearly superior at controlling primary tumor growth when compared to MTD CTX or LDM CTX. (ii) CTX140 1q6d causes some of its anti-tumor effects by stimulating the innate and adaptive immune system (at least in the EMT6/P model). (iii) CTX upregulates PD-L1 expression in vitro and CTX140 1q6d upregulates PD-L1 expression on CD45^+^ and CD45^−^ cells within the tumor microenvironment. (iv) None of the CTX regimens synergized with PD-L1 antibody therapy in the EMT6-CDDP breast cancer model.

CTX140 1q6d reduced primary tumor growth, extended survival, and outperformed MTD CTX and continuous LDM CTX in all three immunocompetent breast tumor models tested. The therapeutic effects of the CTX140 1q6d protocol in EMT6/P are likely due to a mixture of direct cytotoxic effects on tumor cells, as well as enhanced tumor cell killing mediated by NK cells and CD8^+^ T cells. In contrast, in the SP1-AC2M2 breast cancer model, there is a more pronounced cytotoxic effect of CTX (Fig. 2a) as these tumor cells are significantly more sensitive to this chemotherapy agent (Supplementary Fig. 8). Additionally, the SP1-AC2M2 model showed significant responsiveness to all treatment regimens of CTX when survival is compared to control. However, due to the regrowth of tumor re-challenges in mice that displayed complete regressions in both CTX140 1q6d and MTD CTX regimens, we can rule out the development of tumor host immunity in this model. As memory T cells are thought to be maintained by intermittent proliferation^38^ it is possible that quasi-weekly CTX administration depletes these cells. This is indeed the case in the EMT6-CDDP primary tumor model when treated with all CTX regimens, especially in CTX140 1q6d where CD4^+^ effector memory T cells (CD4^+^ CD44^+^ CD62L^−^) were reduced to a third of control treated and CD8^+^ effector memory T cells were reduced by roughly two thirds of control treatment (Supplementary Fig. 3). CTX reducing the population of CD8^+^ central memory T cells has been shown previously with daily doses of CTX at 50 mg/kg in mice^39^. Another study also showed 50% reductions in memory T cells in the spleen as a result of CTX administration, although memory T cells in bone marrow were unaffected^40^. Additionally, in a previous study by Wu and Waxman^19^ when the CTX140 1q6d protocol was used in the treatment of GL261 subcutaneous gliomas in immunocompetent mice, a third of these mice did not display immunity to GL261 re-challenge after complete tumor responses were achieved, reinforcing that adaptive memory is not induced in all cases of complete regression in this model. Our finding that five doses of CTX140 1q6d in the SP1-AC2M2 model followed by treatment break is insufficient to bring about complete responses (Fig. 2d) suggests that continuous pressure of CTX140 1q6d is needed in this model.

In the EMT6-CDDP primary tumor model, CTX140 1q6d reduced granulocytes and increased monocytes, which could potentially mean a switch in granulocytic myeloid-derived suppressor cells (MDSCs) to monocytic MDSCs. Previous reports by others^41^ in models of lymphoma have shown increases in monocytic MDSCs due to CTX treatment resulting in immunosuppression and, this may explain the tumor escape we observed that is later seen in EMT6-CDDP treated with CTX140 1q6d. CyTOF analysis also revealed an increase in CD4^+^ and CD8^+^ T cells, as well as, B cells. However, analysis at this time point (i.e. 6 days after the last CTX140 dose) allows cells to recover from CTX over 6 days; therefore, it gives us an estimation of cell types present before another dose of CTX140 is administered. It is probable that these patterns of increased or decreased immune cells are completely different when analyzed directly after CTX140 administration.

Postsurgical adjuvant treatment of EMT6-CDDP tumor-bearing mice using both MTD CTX and the CTX140 1q6d protocol outperformed control and LDM CTX; however, CTX140 1q6d had no significant benefit over MTD CTX in this treatment setting. When CTX140 1q6d treatment was continued there was a greater significant benefit on survival than CTX140 1q6d (five doses) compared with control, suggesting that CTX140 1q6d treatment needs to be maintained longer than five doses to effectively prolong survival. Each CTX regimen, especially MTD CTX, and each CTX140 1q6d treatment groups effectively inhibited the formation of lung metastases, compared to control-treated mice where over 85% of mice displayed lung metastases. This suggests that CTX has strong anti-metastatic activity even in the MTD and LDM CTX treatment groups.

Here we show that CTX has the capacity to increase expression of PD-L1 on the tumor cell surface. In clinical trials targeting PD-1 or PD-L1 in TNBC patients, those who expressed higher levels of PD-L1 in the tumor microenvironment experienced better responses; for this reason, evaluating the combination of CTX with PD-L1 blockade seemed like a logical pursuit. However, PD-L1 blockade with concomitant CTX treatment or CTX140 1q6d induction treatment failed to synergize in primary tumor models of EMT6-CDDP. As the PD-L1 antibody clone 6E11 contains mutations inhibiting its binding to activating Fcγ receptors, it cannot elicit antibody-dependent cellular cytotoxicity. This was engineered to avoid depletion of PD-L1-expressing immune cells such as dendritic cells or activated T cells important for an anti-tumor response^42,43^. Hence, this is a preclinical surrogate of the PD-L1 targeting antibody atezolizumab which is approved for TNBC in combination with nab-paclitaxel^2,44^. However, it is possible that other PD-L1 antibodies that can bind to Fc receptors may have increased effects in combination with CTX especially as this chemotherapy increases PD-L1 expression on tumor cells. A phase II clinical trial (TONIC) result has recently been published which involved investigating treatment of metastatic TNBC in which low-dose metronomic chemotherapy regimens were administered as induction strategies to test whether they improve follow-up with nivolumab (a PD-1 antibody) therapy. In this trial, low-dose CTX (50 mg daily) induction therapy did not improve responses compared to patients not receiving induction therapy but receiving nivolumab. In fact, patients receiving CTX induction therapy had reductions in objective response rates (ORRs) compared to no induction therapy, 8% and 17%, respectively. However, induction treatment with metronomic doxorubicin or cisplatin did increase ORRs, namely by 35% and 23%, respectively^45^. Although this clinical study had low patient numbers it is somewhat consistent with what we have observed in our preclinical TNBC models showing no increased efficacy when CTX is combined with PD-L1 blockade; however, additional preclinical models and clinical investigations will need to be performed to determine whether CTX can be considered a partner with PD-1/PD-L1 inhibition.

Despite these negative findings involving combination with PD-L1 antibodies, the CTX140 1q6d treatment regimen may be compatible for combining with other immunotherapies; for example, in preclinical models of lymphoma, CTX was found to successfully synergize with blockade of the “don’t eat me” signal CD47, which resulted in enhanced dendritic cell antigen presentation and T cell-mediated clearance of A20 lymphoma cells^46^. Interestingly, in this model if mice were treated with CTX before CD47 blockade, they were resistant to tumor re-challenge; however, if CTX was administered after CD47 antibodies, tumor immune memory was impaired and tumor re-challenges grew. This could reinforce findings of our study where complete regressions in the SP1-AC2M2 model did not protect mice from tumor re-challenge. Similar findings have shown that CTX given after CTLA-4 blockade in a CT26 colon model can attenuate its effects by causing apoptosis of highly proliferative tumor specific CD8^+^ T cells^47^. It is also worth noting that CTX in combination with PD-L1 antibody blockade using the 6E11 antibody clone has been shown to have increased efficacy in preclinical models of MC38 colorectal cancer; therefore, it is possible that this combination therapy may be suitable for other tumor types other than breast cancer^48^. However, in this study, the authors did not re-challenge mice that displayed complete regressions as a result of combination therapy, so it cannot be concluded whether CTX with PD-L1 blockade induced elevated tumor host immunity in this model.

Further work will need to be carried out to determine whether CTX is a promising combination partner for various other immunotherapy treatment strategies, or whether depletion of memory T cells will impair the long-lasting effects of any potential complete responses. Nevertheless, the CTX140 1q6d (or perhaps a similar regimen given weekly) may be a promising option to consider for controlling tumor growth even as a monotherapy and the human equivalent of the protocol may be an attractive therapy for maintenance or palliative care therapy for breast cancer, despite the potential of being unsuitable for combination with immunotherapies targeting PD-L1. Some have argued that the currently used regimen of CTX administration, i.e., MTD or indeed the 50 mg daily oral metronomic dose (which is the closest equivalent to our 20 mg/kg dose of LDM CTX in mice), is sub-optimal for use in cancer patients^21,49^. A suggested dose of 7.3–11.4 mg/kg or 270–420 mg/m^2^ has been proposed as a human equivalent when based on body surface area calculations from mice to humans^21^. With data presented here as well as other clinical evidence suggesting that daily low-dose CTX (50 mg daily) may not be an optimal treatment in breast cancer (at least when combined with docetaxel)^50^, this begs the question whether a “medium dose” of CTX should be tested in clinical trials. If found to be effective, the use of such an off-patent, inexpensive chemotherapy drug may be of particular benefit in low-income countries that cannot afford the excessively high cost of new targeted therapeutics—including immune checkpoint antibodies^51^.

## Methods

### Cell lines

All cell lines were cultured in Dulbecco’s modified Eagle’s medium with 5% FCS. All cell lines used were regularly tested and free of mycoplasma (Mycoalert Kit; Lonza). EMT6/P (EMT6-parental) and EMT6-CDDP (EMT6-cisplatin resistant) were originally from Dr. Beverly Teicher^32^. SP1-AC2M2 was obtained from Dr. Bruce Elliot (Queen’s University, Kingston Ontario) as described^30,31^.

### Primary tumor studies

Animal experiments were carried out with the approval of the institutional Animal Care Committee in accordance to Canadian Council on Animal Care (CCAC) guidelines. Female BALB/c, CBA/J, and SCID CB17 mice were purchased from Jackson Laboratories. Female SCID Beige (CB17.Cg-*Prkdc*^*scid*^*Lyst*^*bg-J*^/Crl) mice were purchased from Charles River Laboratories. Female BALB/c mice aged 6–8 weeks were implanted in the inguinal mammary fat pad with 100,000 cells of EMT6/P or EMT6-CDDP. Female CBA/J mice aged 6–8 weeks were implanted in the inguinal mammary fat pad with 50,000 SP1-AC2M2 cells.

### In vivo therapies

Primary tumor treatment was initiated when tumors were an average of 150–250 mm^3^ depending on the model; mice were separated into groups ensuring the mean tumor volume was as similar as possible in each group. CTX (Procytox, Baxter) was reconstituted at 20 mg/mL in normal sterile saline and further diluted as needed. MTD CTX was administered as 150 mg/kg i.p. injection followed by two doses of 100 mg/kg every other day. This cycle was repeated after 3 weeks. LDM daily CTX was administered in the drinking water for an estimated dose of 20 mg/kg as previously described, after a bolus dose of 150 mg/kg i.p.^11^. CTX140 1q6d was administered by i.p. injection at 140 mg/kg every 6 days. PD-L1 antibody therapy (clone 6E11; Genentech) was administered at 5 mg/kg by i.p. injection twice every week. Mouse Isotype control antibody (MOPC-21) was administered at 5 mg/kg i.p. twice weekly also (cat. no. BE0083, BioXCell). All antibodies were diluted in sterile phosphate-buffered saline (PBS).

### Postsurgical metastasis studies

Mice implanted with 100,000 EMT6-CDDP cells were monitored for tumor growth by caliper measurements. Upon reaching an average of 200 mm^3^ primary tumors were resected, and mice were split into treatment groups based on average tumor size at resection and other parameters such as how much a tumor had invaded or become attached to the peritoneal wall, to result in a fair representation between all treatment groups. Treatment was initiated 2 days after surgical resection as adjuvant therapy. Mice were monitored daily and sacrificed upon development of labored breathing, loss of more than 20% body weight, or when tumor regrowth became ulcerated or at size limit.

### 4H-CTX in vitro studies

For IC_50_ determination, 3000 cells were plated per well in a 96-well plate and treated with 4-hydroperoxy cyclophosphamide (4H-CTX) (cat. no. H714675, Toronto Research Chemicals) for 48 h and then cell viability was assessed using Deep Blue Cell Viability kit (cat. no. 424701, BioLegend). Fluorescence intensity was measured at excitation 530 nm and emission 590 nm on a BioTek plate reader.

For flow cytometry analysis of PD-L1 expression, each cell type was treated with 4H-CTX or paclitaxel for 48 h and then washed with PBS, detached from plates with trypsin and analyzed by flow cytometry as described below.

### Flow cytometry

For experiments analyzing PD-L1 expression in cell lines, anti-mouse PD-L1 APC (rat clone 10F.9G2) (cat. no. 124312, BioLegend) was used alongside separate stains using Isotype control APC (rat clone RTK4530) (cat. no. 400612, BioLegend). Antibodies were used at a dilution of 1:100 or 2 μg/mL. DAPI was used to stain dead cells (200 ng/mL) and these were excluded upon analysis.

For analysis of in vivo PD-L1 expression, tumors were dissociated with the following enzymatic buffer (1% BSA, 12,500 units collagenase II (Worthington), 12,500 units collagenase IV (Worthington), and DNase I (cat. no. LS006333, Worthington) for 45 min and then a single-cell suspension gained. After multiple washes with PBS, cells were then incubated with Zombie Violet fixable viability dye (cat. no. 423114, BioLegend). Then Fc receptors were blocked using FcR block anti-CD32/CD16 for 30 min at 1:100 or 5 μg/mL (cat. no. 553142, BD Biosciences). Then one million cells were stained with the following panel CD45-APC-Cy7 (cat. no. 557659, BD Biosciences), CD11c-AF700 (cat. no. 117319, BioLegend), B220-APC (cat. no. 103211; BioLegend), GR-1-BV650 (cat. no. 108441, BioLegend), CD31 PE-Cy7, PD-L1-PE (cat. no. 124308, BioLegend), CD11b-PerCP-Cy5.5 (cat. no. 550993, BD Biosciences), MHC Class II-I-A/I-E FITC (cat. no. 107605, BioLegend). Flow cytometry was performed on an LSR II (BD Biosciences) and analyzed using FlowJo. Gating strategies are detailed in Supplementary Fig. 6.

### CyTOF acquisition and analysis

The mass cytometry acquisition and analysis were performed as previously described^52,53^. Briefly, BALB/c mice bearing EMT6-CDDP tumors were sacrificed at endpoint (day 29) and tumors were excised and prepared as a single-cell suspension as previously described^54^. Samples from each tumor of the same group were pooled (*n* = 4–5 tumors/group), and four million cells were stained with a mixture of metal-tagged antibodies. Metal tags as detailed in Supplementary Table 1 using the following antibodies: CD45 (cat. no. 103120, BioLegend), CD80 (cat. no. 104702, BioLegend), GR1 (cat. no. 108402, BioLegend), CD86 (ca, no. 105002, BioLegend), F4/80 (cat. no. 123102, BioLegend), CD4 (cat. no. 100520, BioLegend), CD45R (cat. no. 103202, BioLegend), Ly6c (cat. no. 128002, BioLegend), CD138 (cat. no. 149002, BioLegend), CD8 (cat. no. 100716, BioLegend), Ly6g (cat. no. 128002, BioLegend), CD206 (cat. no. 141702, BioLegend), CD25 (cat. no. 101913, BioLegend), CD126 (cat. no. 112702, BioLegend), CD11c (cat. no. 117302, BioLegend), CCR9 (cat. no. 129704, BioLegend), CD49b (cat. no. 103513, BioLegend), CD19 (cat. no. 115502, BioLegend), CD34 (cat. no. 553731, BD Biosciences), CD27 (cat. no. 120101, BioLegend), CD69 (cat. no. 104502, BioLegend), CD150 (cat. no. 115933, BioLegend), TCRb (cat. no. 109202, BioLegend), CD127 (cat. no. 135002, BioLegend), CD28 (cat. no. 102102, BioLegend), CD115 (cat. no. 135502, BioLegend), Siglec-F (cat. no. MAB1706, R&D Systems/Novus Biologicals), CD93 (cat. no. MAB1696, R&D systems/Novus Biologicals), CD117 (cat. no. 105802, BioLegend), CD79b (cat. no. 132802, BioLegend), CD62L (cat. no. 104402, BioLegend), CD44 (cat. no. 103014, BioLegend), CXCR4 (cat. no. 146502, BioLegend), Sca-1 (cat. no. 108102, BioLegend), VEGFR2 (cat. no. 409302, BioLegend), CD5 (cat. no. 100602, BioLegend), CD11b (cat. no. 101202, BioLegend). The antibodies were conjugated with MAXPAR reagent (Fluidigm Inc.) according to the manufacturer’s instructions. Live and dead cells were identified by rhodium and iridium intercalators. After several washing steps, cells were acquired by CyTOF. The analysis was performed using Cytobank software as previously described^52,53^ and viSNE and SPADE algorithms were used. Population was defined as positive above the threshold 10^1^. Raw CyTOF data available online^55^.

### Immunohistochemistry

Upon sacrifice, lungs were inflated with a mixture of 1:1.5 OCT:PBS using a 26G needle. Inflated lungs were then embedded in OCT or FSC 22 Clear Frozen Section compound (cat. no. 3801480, Leica) and snap frozen and stored at −80 °C. Lungs were cryo-sectioned at 12 µm and then stained for expression of Ki67. Sections were allowed to thaw for 10 min, and then fixed in ice cold acetone (5 min). Endogenous peroxidases were quenched with 1% aqueous H_2_O_2_ in methanol for 15 min and then blocked in Dako Blocking reagent (cat. no. X0909, Dako) supplemented with 10% donkey serum (no. 017-000-121, Jackson ImmunoResearch) for 30 min. Ki67 (D3B5) Rabbit mAb (Mouse Preferred; IHC Formulated) (cat. no. 12202, Cell Signalling Technologies) was incubated overnight at 4 °C at a dilution of 1:200 in Dako antibody diluent with background reducing components (cat. no. S3022, Dako). After 3 washes in PBST, sections were incubated with LSAB2 Streptavidin-Peroxidase/SA-HRP kit (DAKO K0690) for 30 min, and then mounted using Permount (cat. no. # SP15-500, Fisher Scientific). Images were acquired on a Leica DM LB2 microscope and DFC 300 FX camera. ImageJ was used to determine the pixel area of the lung and also pixel area of metastatic nodules, from which the percentage metastasis area was calculated.

### Statistical analysis

All statistical analyses were performed using Graphpad Prism Version 5.02. All measurements are from distinct samples, at multiple time points for experiments such as primary tumor growth. All ANOVA and *t*-tests were two-tailed tests. *N* numbers are detailed in figure legends or displayed as individual data points.

### Reporting summary

Further information on research design is available in the Nature Research Reporting Summary linked to this article.

## Supplementary information

Reporting Summary

Supplementary Figures 1-8 + Supplementary Table 1

## Data Availability

The data generated and analyzed during this study are publicly available in the figshare repository, as part of the following data record: 10.6084/m9.figshare.12383498^55^. Datasets supporting the supplementary figures in the published article are available on reasonable request from the corresponding authors, as described in the data record above.
