# Supplementary material for: Immunostimulatory and anti-tumor metronomic cyclophosphamide regimens assessed in primary orthotopic and metastatic murine breast cancer
Source: NPJ Breast Cancer. 2020 Jul 20;6:29. doi: 10.1038/s41523-020-0171-1 (PMC7371860; doi:10.1038/s41523-020-0171-1)
Supplement: Supplementary file 2 — Supplementary Figures 1-8 + Supplementary Table 1 [file 41523_2020_171_MOESM2_ESM.pdf]

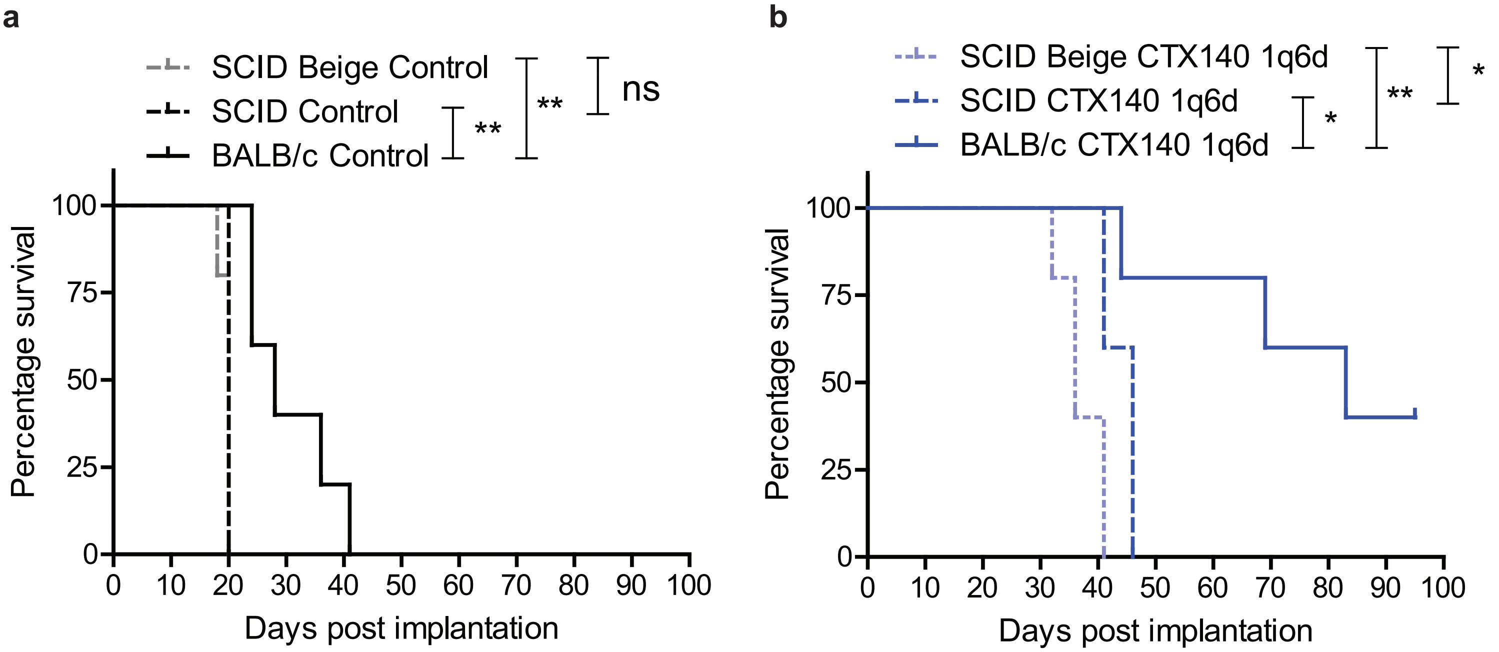


**Supplementary Figure 1. Comparison of immunocompetent BALB/c vs immunodeficient SCID and SCID Beige mice control treated or CTX140 1q6d treated. a** In control treated mice, there is a significant difference between both SCID and SCID Beige when compared to normal BALB/c mice, however there is no significant difference between SCID and SCID beige, indicating that lack of NK cells does not have a major influence on the primary tumor growth in untreated mice. **b** In CTX140 1q6d treated mice there is a significant difference between normal BALB/c and SCID mice suggesting the adaptive immune system is somewhat involved, and there is also a significant difference between SCID and SCID Beige, suggesting that when NK cells are not present as well as T-cells and B-cells, the CTX140 1q6d treatment has a reduced effect on prolonging survival. It is interesting to note that while there is no significant difference between SCID and SCID Beige in control treated mice, there is a difference in survival when treated with CTX140 1q6d. This reinforces that NK cells are involved, but likely T-cells are also important.

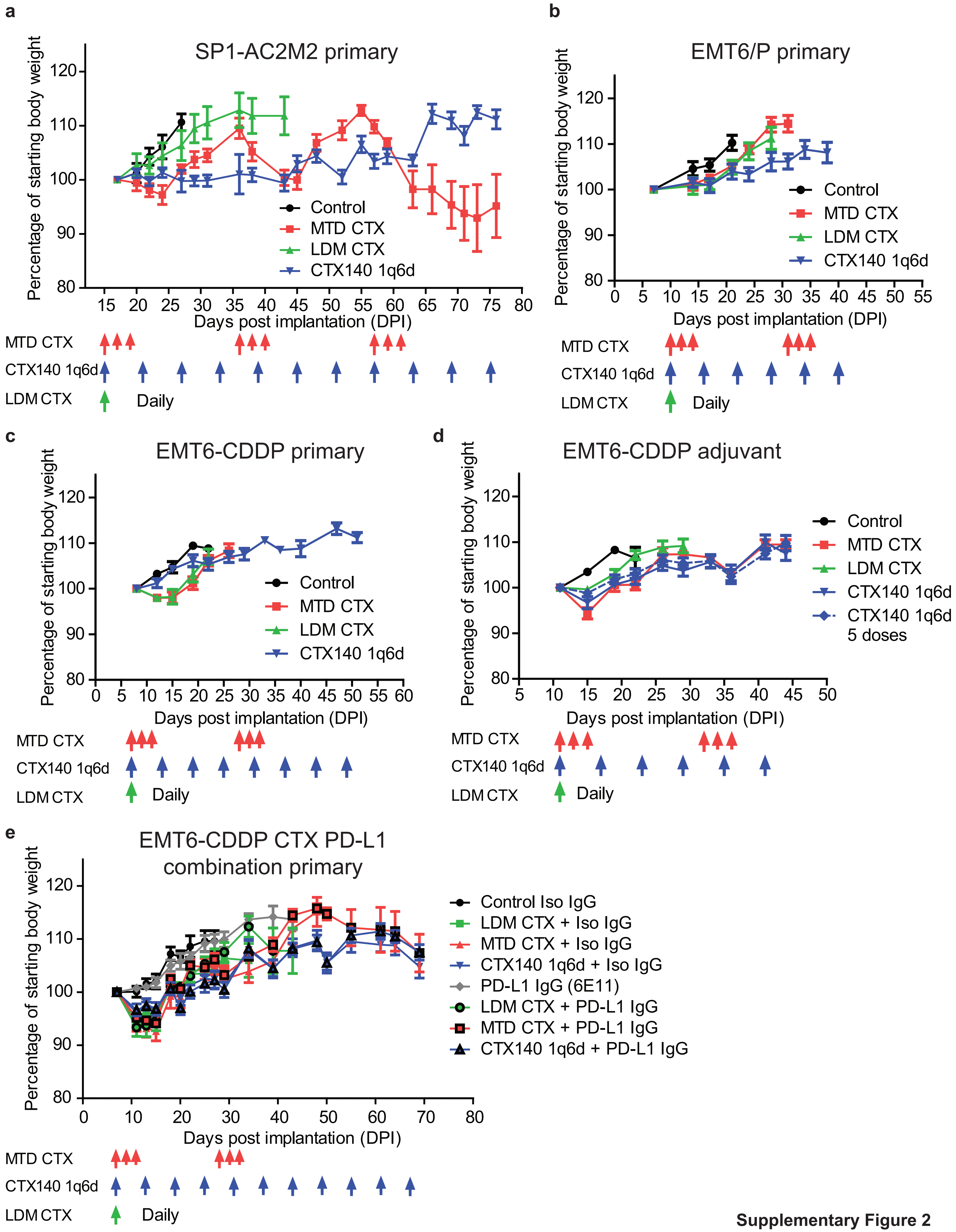


**Supplementary Figure 2**

**Supplementary Figure 2. Mouse body weights from each experiment displayed as a percentage of body weight at start of therapy. a** SP1-AC2M2 primary tumour model, showing that CBA/J mice treated with LDM CTX lose weight after the first CTX dose and then begin to gain weight comparable with control treatment. MTD CTX treated mice lose weight with each cycle of MTD CTX but this recovers in the break period, until the 3rd dose where the average weight reduces ~10% from starting weight. Some mice were euthanized as their weight reduced and they became moribund. CTX140 1q6d treated mice gradually gain weight but not at the same rate as control treated mice. **b** EMT6 primary tumour treatment, showing similar to (A) that body weight increases with time in all CTX treated groups but not at the same rate as control treatment. **c** EMT6-CDDP primary tumour and **d** EMT6-CDDP adjuvant show similar trends as seen with SP1-AC2M2 and EMT6/P models. **e** EMT6-CDDP CTX and PD-L1 combination treatment shows similar patterns to all other models, control IgG and PD-L1 IgG do not result in weight loss as expected. Error bars display SEM.

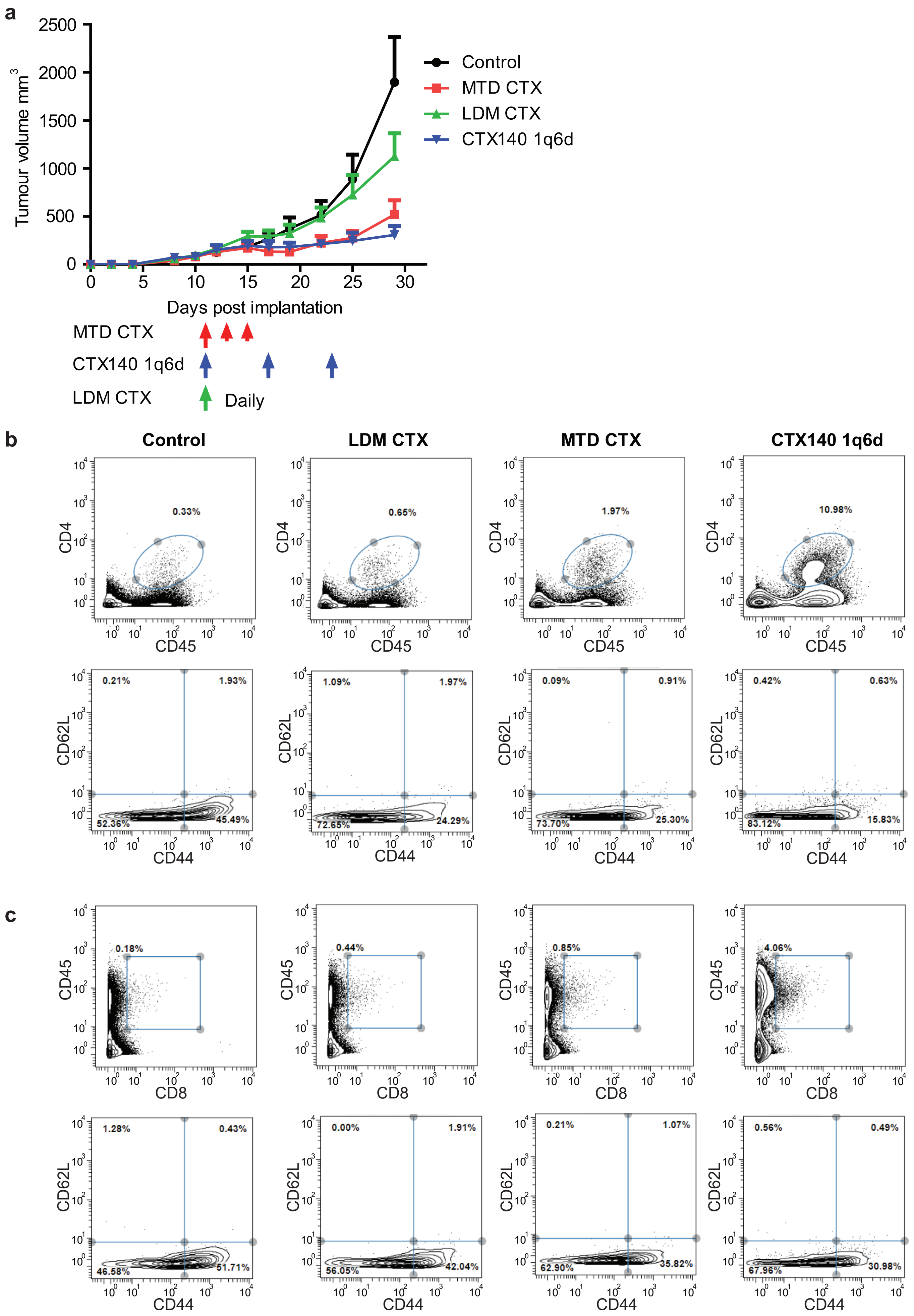


**Supplementary Figure 3**

**Supplementary Figure 3. CyTOF analysis of EMT6-CDDP treated with CTX. a** Primary tumor growth curves of EMT6-CDDP treated with each CTX treatment group. Mice were culled at one time point (29 DPI) for CyTOF analysis. **b** Analysis of CD44 and CD62L memory markers in $CD4^{+}$ T-cells from CyTOF experiment. **c** Analysis of CD44 and CD62L memory markers in $CD8^{+}$ T-cells from CyTOF experiment.

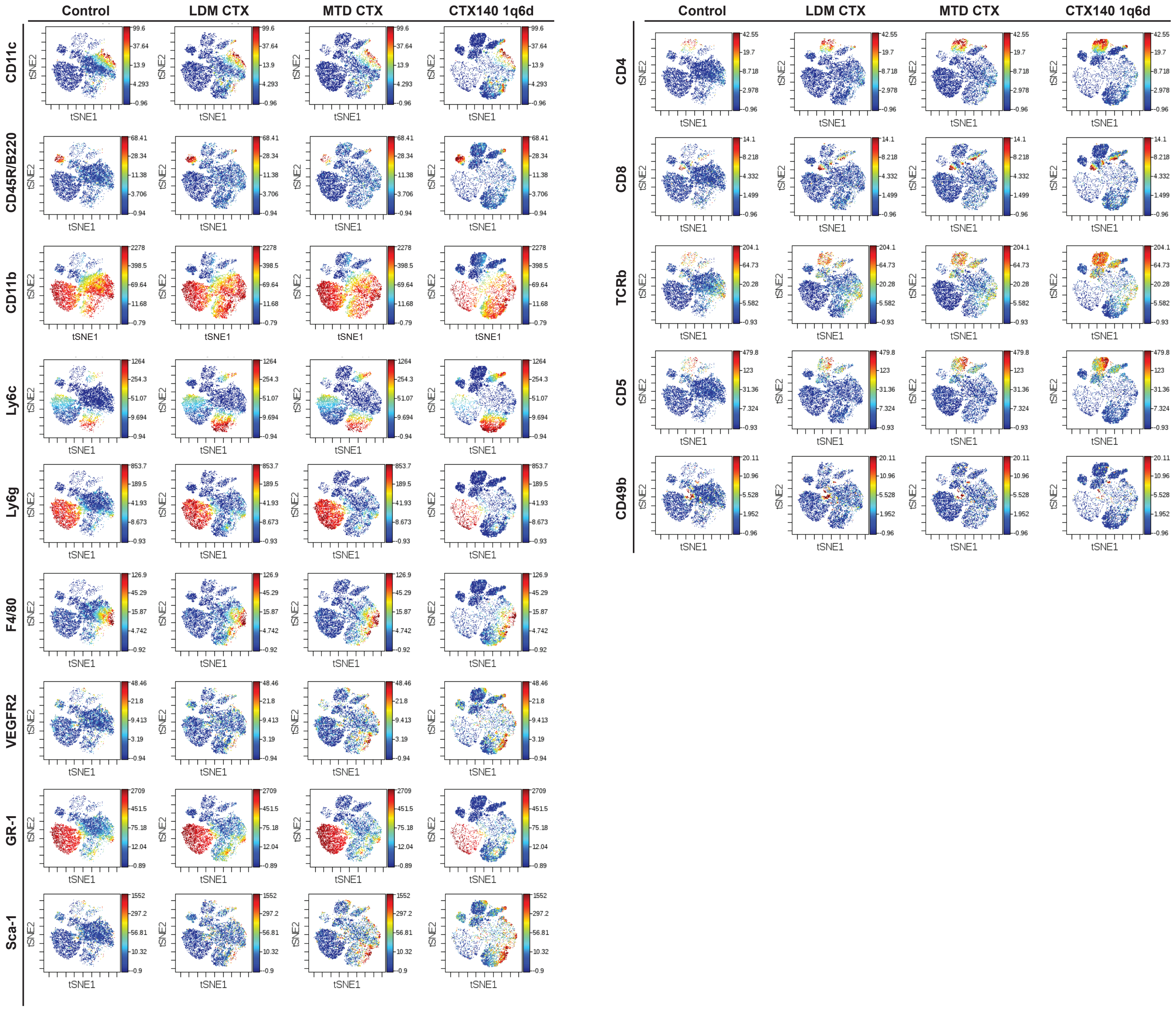


**Supplementary Figure 4. tSNE plots of CyTOF analysis of EMT6-CDDP treated with CTX.** Including the select markers CD11c, CD45R, CD11b, Ly6c, Ly6g, F4/80, VEGFR2, GR-1, Sca-1, CD4, CD8, TCRb, CD5, and CD49b.

**a**

| Mouse | **Saline control** | **LDM CTX** | **MTD CTX** | **CTX140 1q6d continuous** | **CTX140 1q6d 5 doses** |
|---|---|---|---|---|---|
| 1 | ++++ | + | - | - | - |
| 2 | - | - | - | - | - |
| 3 | + | +++ | - | - | - |
| 4 | ++++ | +++ | ++ | - | - |
| 5 | ++++ | - | - | - | - |
| 6 | ++++ | ++ | - | - | - |
| 7 | ++++ | - | - | - | - |
| 8 | ++++ | - | ++ | - | +++ |

**b**

| **Treatment group** | **Median Survival DPI** | ***$P_{log\text{-}rank}$ vs Control*** | ***$P_{log\text{-}rank}$ vs MTD CTX*** | ***$P_{log\text{-}rank}$ vs LDM CTX*** | ***$P_{log\text{-}rank}$ vs CTX140 1q6d*** | ***$P_{log\text{-}rank}$ vs CTX140 1q6d (5 doses)*** | **HR *vs Control* (95% CI)** |
|---|---|---|---|---|---|---|---|
| Control | 31 | - | 0.0023** | 0.0933 ns | 0.0033** | 0.0142* | - |
| MTD CTX | Undefined | 0.0023** | - | 0.1038 ns | 0.7489 ns | 0.2091 ns | 0.103 (0.024-0.443) |
| LDM CTX | 53 | 0.0933 ns | 0.1038 ns | - | 0.1838 ns | 0.6484 ns | 0.353 (0.105-1.191) |
| CTX140 1q6d | Undefined | 0.0033** | 0.7489 ns | 0.1838 ns | - | 0.2034 ns | 0.119 (0.028-0.493) |
| CTX140 1q6d (5 doses) | 59 | 0.0142* | 0.2091 ns | 0.6484 ns | 0.2034 ns | - | 0.188 (0.050-0.715) |

**c**

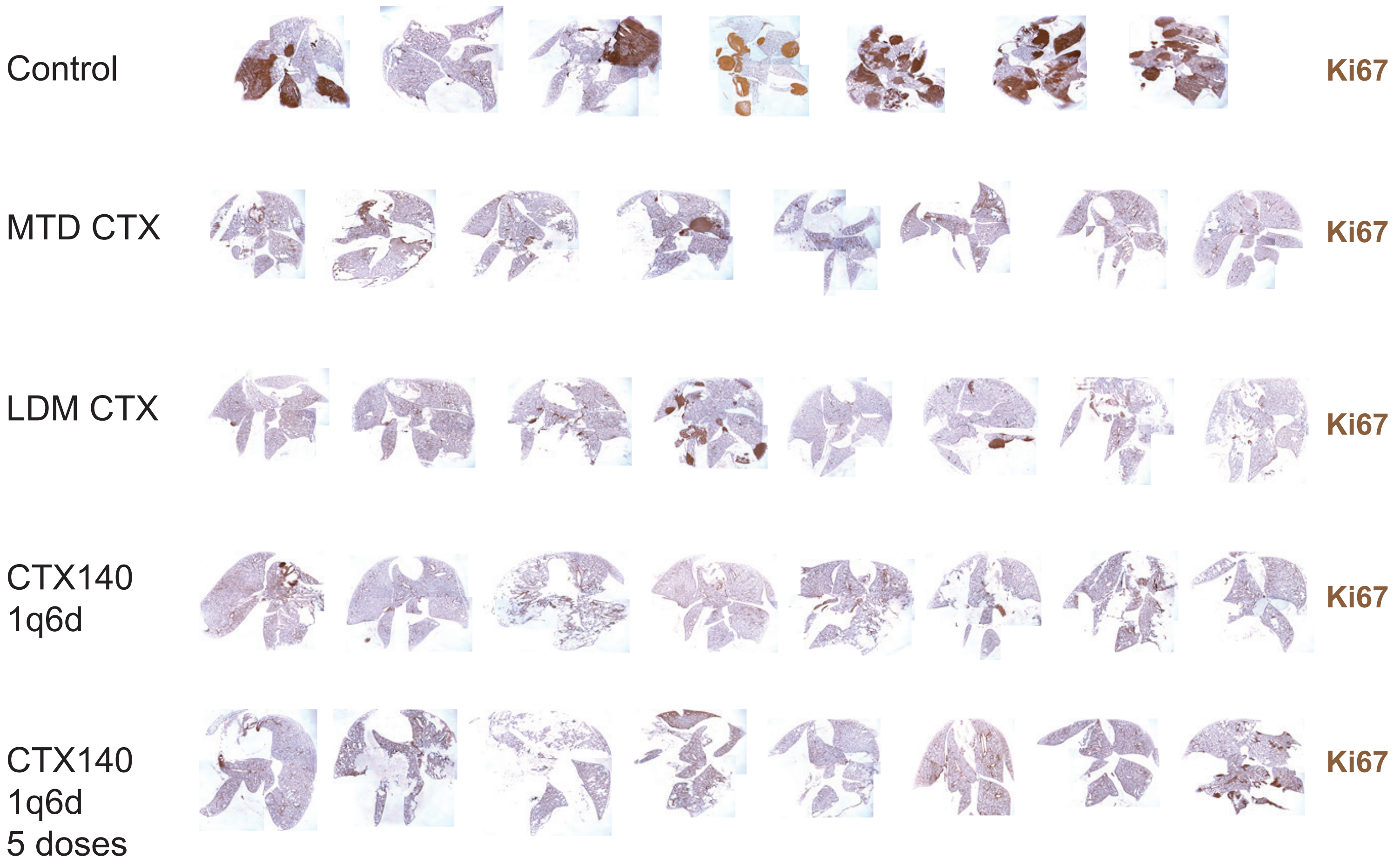


**Supplementary Figure 5**

**Supplementary Figure 5. Lung metastases in the EMT6-CDDP adjuvant treatment experiment. a** Table of macroscopic lung metastasis visualised upon necropsy. Each + denotes whether macroscopic lung metastases were observed and to what degree, + = low burden, ++ = medium burden, +++ = high burden, ++++ = very high burden, and - = signifies no observable lung macrometastases. **b** Table of Log rank Mantel Cox statistical survival analyses of EMT6-CDDP adjuvant treatment. **c** Microscope images of lungs stained for Ki67 expression used in the analysis of Figure 4c. Debris and other organs such as heart were removed from some images for clarity.

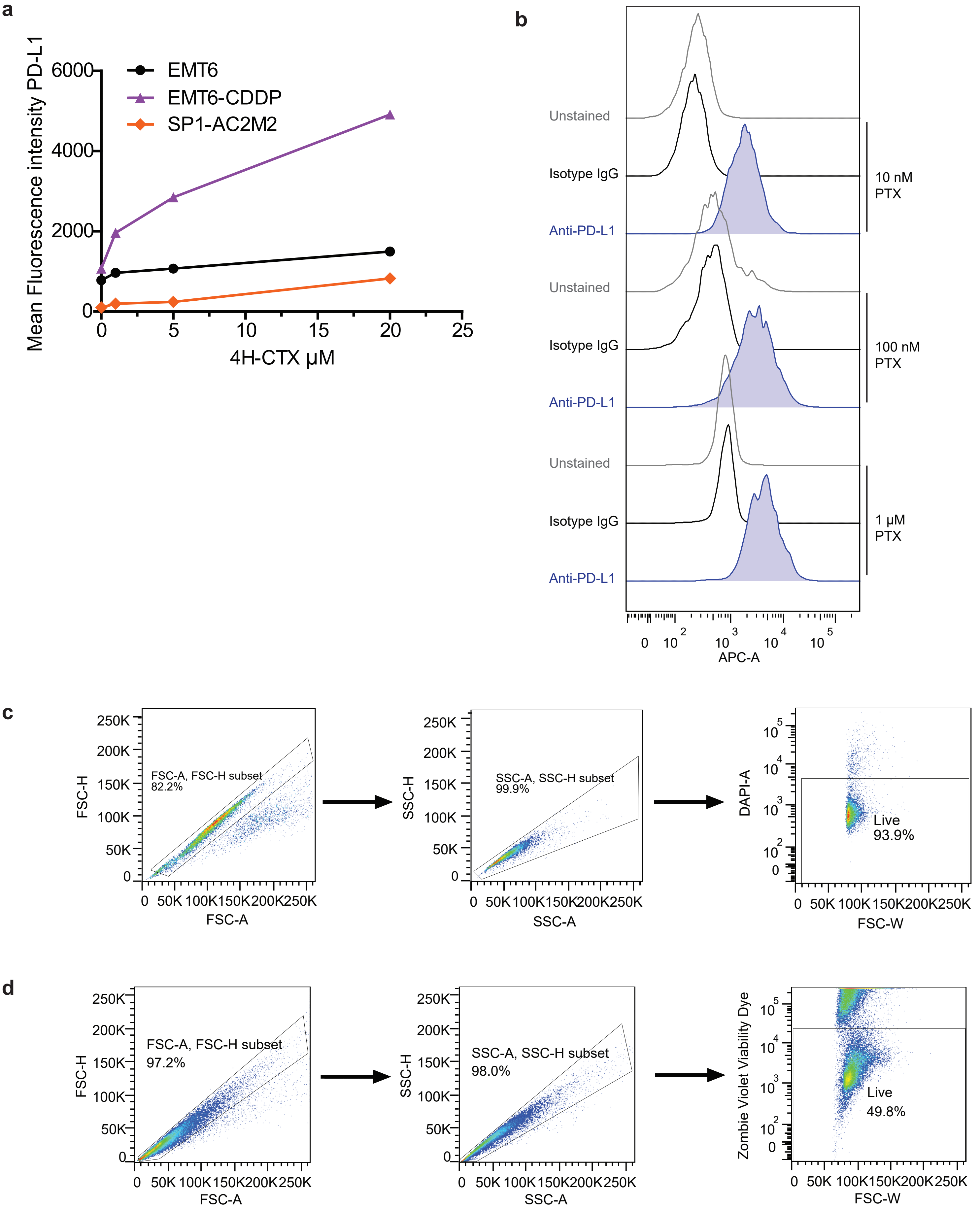


**Supplementary Figure 6**

**Supplementary Figure 6. EMT6/P, EMT6-CDDP and SP1-AC2M2 4H-CTX treatments. a** All cell lines show upregulation of PD-L1 in a dose dependent manner in response to 4H-CTX. **b** Representative histogram plots of EMT6-CDDP cells treated with paclitaxel (PTX) as a positive control for PD-L1 upregulation. **c** Representative plots showing gating strategy of experiments from Figure 5. **d** Representative plots showing gating strategy of experiments from Supplementary Figure 7.

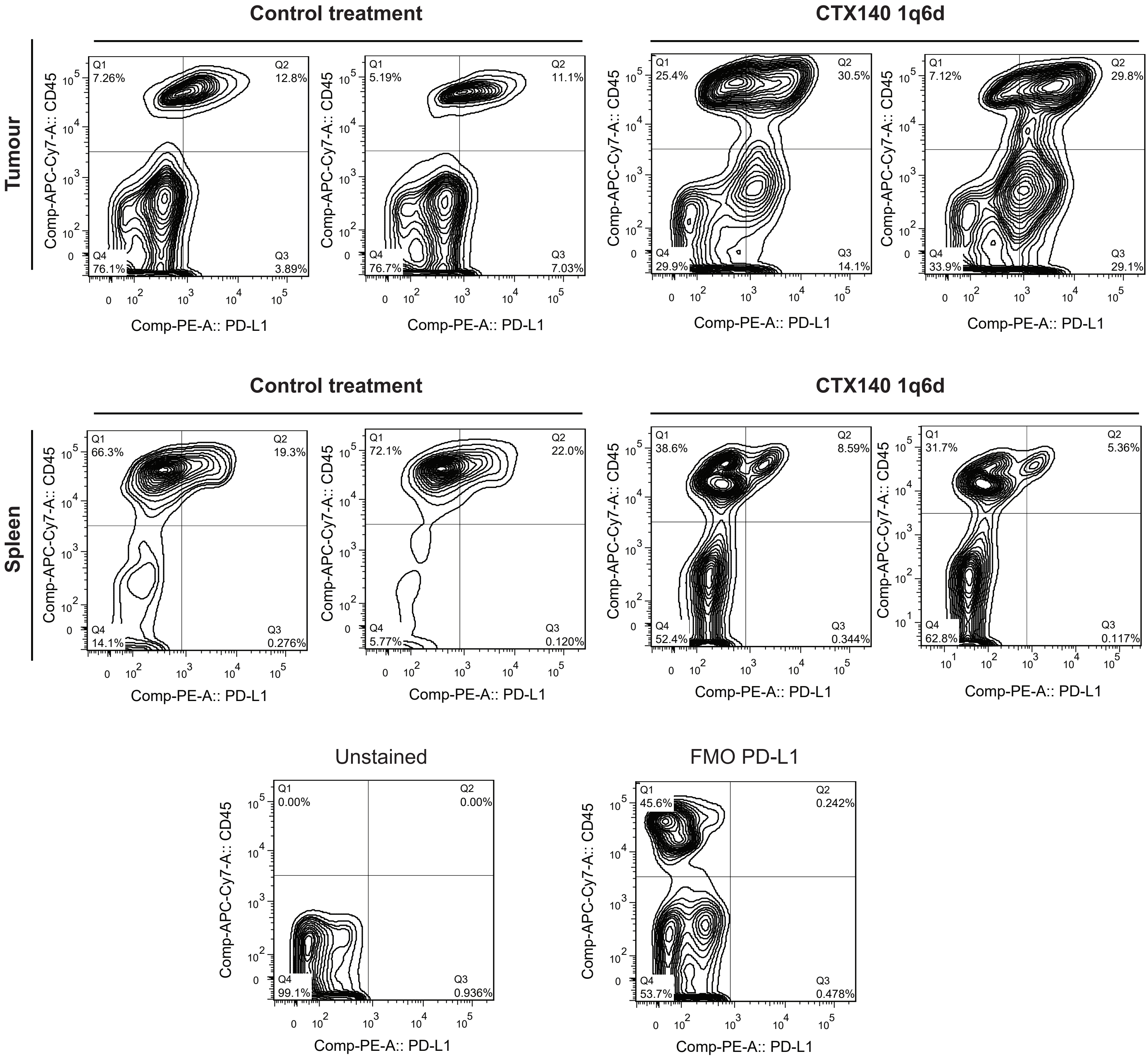


**Supplementary Figure 7. CTX140 1q6d increases PD-L1 expression within SP1-AC2M2 primary tumor but reduces expression in spleen.** CD45$^+$ PD-L1$^+$ and CD45$^-$ PD-L1$^+$ cells within SP1-AC2M2 tumors increase in two mice treated with 3 doses of CTX140 1q6d in comparison to two mice treated with saline control. However, CD45$^+$ PD-L1$^+$ cells decrease in spleens when mice are treated with CTX140 1q6d. Unstained and fluorescence minus one (FMO) for PD-L1 staining is shown to demonstrate gating strategy.

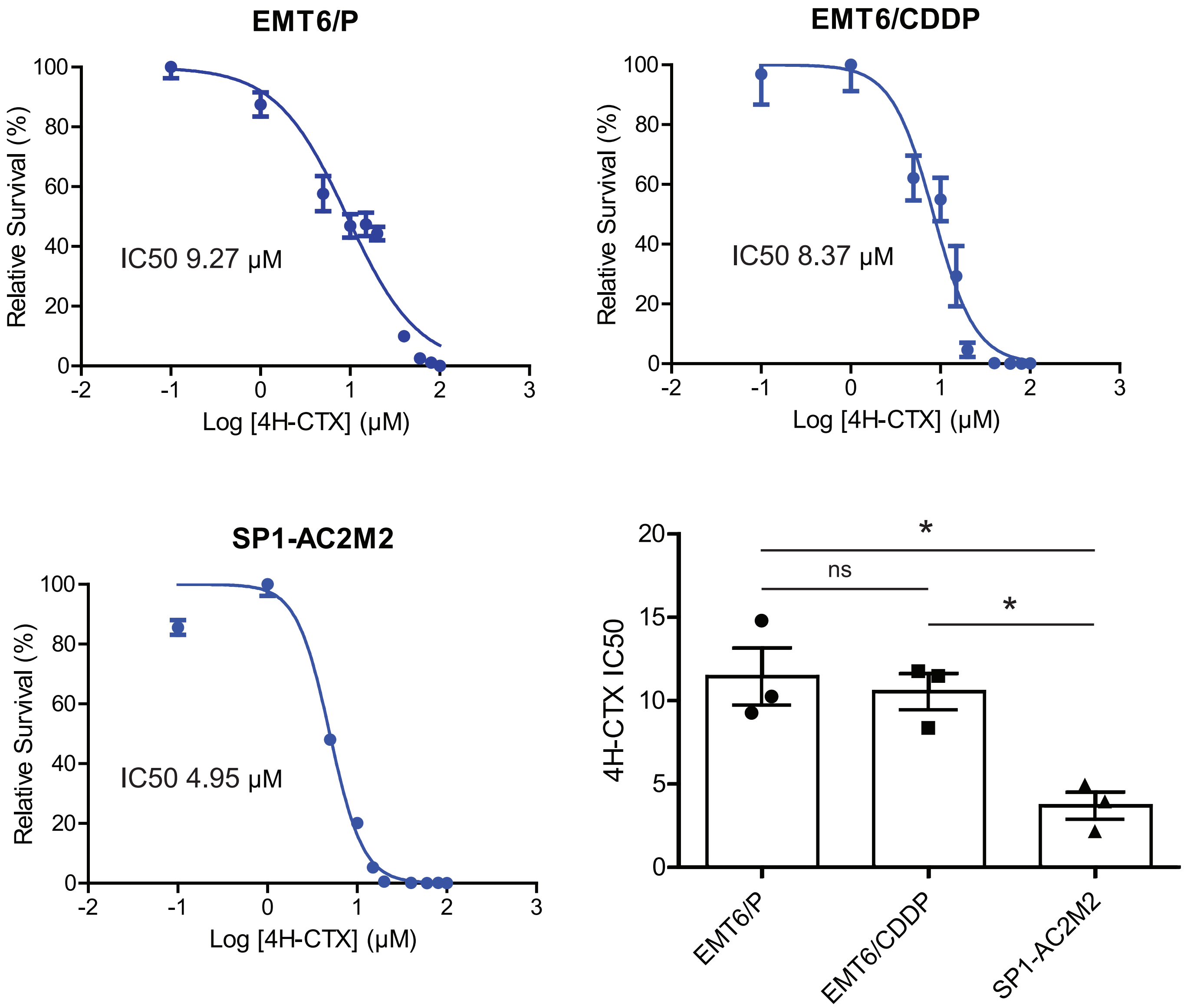


**Supplementary Figure 8. 4H-CTX IC50 of EMT6/P, EMT6-CDDP and SP1-AC2M2.** SP1-AC2M2 is significantly more sensitive in vitro to 4H-CTX than EMT6/P or EMT6-CDDP. One-way analysis of variance with Tukey's multiple comparison test. $^*p<0.05$ $n=3$.

| # | Metal | Marker | Clone | Supplier | Cat no. |
|---|---|---|---|---|---|
| 1 | 115In | CD45 | 30-F11 | Biolegend | 103120 |
| 2 | 141Pr | CD80 | 16-10A1 | Biolegend | 104702 |
| 3 | 142Nd | GR1 | RB6-8C5 | Biolegend | 108402 |
| 4 | 143Nd | CD86 | GL-1 | Biolegend | 105002 |
| 5 | 144Nd | F4/80 | BM8 | Biolegend | 123102 |
| 6 | 145Nd | CD4 | RM4-5 | Biolegend | 100520 |
| 7 | 146Nd | CD45R | RA3-6B2 | Biolegend | 103202 |
| 8 | 147Sm | Ly6c | HK1.4 | Biolegend | 128002 |
| 9 | 148Nd | CD138 | SA011F11 | Biolegend | 149002 |
| 10 | 149Sm | CD8 | 53-6.7 | Biolegend | 100716 |
| 11 | 150Nd | Ly6g | 1A8 | Biolegend | 128002 |
| 12 | 151Eu | CD206 | C068C2 | Biolegend | 141702 |
| 13 | 152Sm | CD25 | 3C7 | Biolegend | 101913 |
| 14 | 153Eu | IL-6r (CD126) | 1B1.3a | Biolegend | 112702 |
| 15 | 154Sm | CD11c | N418 | Biolegend | 117302 |
| 16 | 155Gd | CCR9 | 9B1 | Biolegend | 129704 |
| 17 | 156Gd | CD49b | HMα2 | Biolegend | 103513 |
| 18 | 157Gd | CD19 | 6D5 | Biolegend | 115502 |
| 19 | 158Gd | CD34 | RAM34 | BD Biosciences | 553731 |
| 20 | 159Tb | CD27 | LG.3A10 | Biolegend | 120101 |
| 21 | 160Gd | CD69 | H1.2F3 | Biolegend | 104502 |
| 22 | 161Dy | CD150 | TC15-12F12.2 | Biolegend | 115933 |
| 23 | 162Dy | TCRb | H57-597 | Biolegend | 109202 |
| 24 | 163Dy | CD127 | A7R34 | Biolegend | 135002 |
| 25 | 164Dy | CD28 | 37.51 | Biolegend | 102102 |
| 26 | 165Ho | CD115 | AFS98 | Biolegend | 135502 |
| 27 | 166Er | SiglecF | 238023 | R&D Systems | MAB1706 |
| 28 | 167Er | CD93 | 223437 | R&D Systems | MAB1696 |
| 29 | 168Er | CD117 | 2B8 | Biolegend | 105802 |
| 30 | 169Tm | CD79b | HM79-12 | Biolegend | 132802 |
| 31 | 170Er | CD62L | MEL-14 | Biolegend | 104402 |
| 32 | 171Yb | CD44 | IM7 | Biolegend | 103014 |
| 33 | 172Yb | CXCR4 | L276F12 | Biolegend | 146502 |
| 34 | 173Yb | Sca-1 | D7 | Biolegend | 108102 |
| 35 | 174Yb | Vegfr2 | HP6017 | Biolegend | 409302 |
| 36 | 175Lu | CD5 | 53-7.3 | Biolegend | 100602 |
| 37 | 176Yb | CD11b | M1/70 | Biolegend | 101202 |

**Supplementary Table 1**
